# Chapter 17: Bioimage Informatics for Systems Pharmacology

**DOI:** 10.1371/journal.pcbi.1003043

**Published:** 2013-04-25

**Authors:** Fuhai Li, Zheng Yin, Guangxu Jin, Hong Zhao, Stephen T. C. Wong

**Affiliations:** NCI Center for Modeling Cancer Development, Department of Systems Medicine and Bioengineering, The Methodist Hospital Research Institute, Weil Medical College of Cornell University, Houston, Texas, United States of America; Whitehead Institute, United States of America; University of Maryland, Baltimore County, United States of America

## Abstract

Recent advances in automated high-resolution fluorescence microscopy and robotic handling have made the systematic and cost effective study of diverse morphological changes within a large population of cells possible under a variety of perturbations, e.g., drugs, compounds, metal catalysts, RNA interference (RNAi). Cell population-based studies deviate from conventional microscopy studies on a few cells, and could provide stronger statistical power for drawing experimental observations and conclusions. However, it is challenging to manually extract and quantify phenotypic changes from the large amounts of complex image data generated. Thus, bioimage informatics approaches are needed to rapidly and objectively quantify and analyze the image data. This paper provides an overview of the bioimage informatics challenges and approaches in image-based studies for drug and target discovery. The concepts and capabilities of image-based screening are first illustrated by a few practical examples investigating different kinds of phenotypic changes caEditorsused by drugs, compounds, or RNAi. The bioimage analysis approaches, including object detection, segmentation, and tracking, are then described. Subsequently, the quantitative features, phenotype identification, and multidimensional profile analysis for profiling the effects of drugs and targets are summarized. Moreover, a number of publicly available software packages for bioimage informatics are listed for further reference. It is expected that this review will help readers, including those without bioimage informatics expertise, understand the capabilities, approaches, and tools of bioimage informatics and apply them to advance their own studies.

**What to Learn in This Chapter**

- What automated approaches are necessary for analysis of phenotypic changes, especially for drug and target discovery?
- What quantitative features and machine learning approaches are commonly used for quantifying phenotypic changes?
- What resources are available for bioimage informatics studies?

This article is part of the “Translational Bioinformatics" collection for *PLOS Computational Biology*.

## 1. Introduction

The old adage that a picture is worth a thousand words certainly applies to the identification of phenotypic variations in biomedical studies. Bright field microscopy, by detecting light transmitted through thin and transparent specimens, has been widely used to investigate cell size, shape, and movement. The recent development of fluorescent proteins, e.g., green fluorescent protein and its derivatives [1], enabled the investigation of the phenotypic changes of subcellular protein structures, e.g., chromosomes and microtubules, revolutionizing optical imaging in biomedical studies. Fluorescent proteins are bound to specific proteins that are uniformly located in relevant cellular structures, e.g., chromosomes, and emit longer wavelength light, e.g., green light, after exposure to shorter wavelength light, e.g., blue light. Thus, the spatial morphology and temporal dynamic activities of subcellular protein structures can be imaged with a fluorescence microscope - an optical microscope that can specifically detect emitted fluorescence of a specific wavelength [2]. In current image-based studies, five-dimensional (5D) image data of thousands of cells (cell populations) can be acquired: spatial (3D), time lapse (1D), and multiple fluorescent probes (1D).

With advances to automated high-resolution microscopy, fluorescent labeling, and robotic handling, image-based studies have become popular in drug and target discovery. These image-based studies are often referred to as the High Content Analysis (HCA) [3], which focuses on extracting and analyzing quantitative phenotypic data automatically from large amounts of cell images with approaches in image analysis, computation vision and machine learning [3], [4]. Applications of HCA for screening drugs and targets are referred to as High Content Screening (HCS), which focuses on identifying compounds or genes that cause desired phenotypic changes [5]–[7]. The image data contain rich information content for understanding biological processes and drug effects, indicate diverse and heterogeneous behaviors of individual cells, and provide stronger statistical power in drawing experimental observations and conclusions, compared to conventional microscopy studies on a few cells. However, extracting and mining the phenotypic changes from the large scale, complex image data is daunting. It is not feasible to manually analyze these data. Hence, bioimage informatics approaches were needed to automatically and objectively analyze large scale image data, extract and quantify the phenotypic changes to profile the effects of drugs and targets.

**Figure 1:**
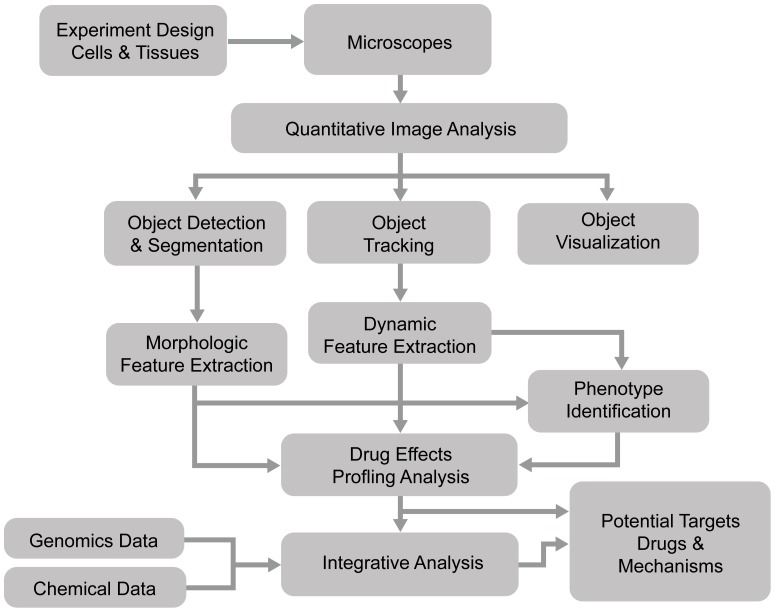
The flowchart of bioimage informatics for drug and target discovery.

Bioimage informatics in image-based studies usually consists of multiple analysis modules [3], [8], [9], as shown in Figure 1. Each of the analysis tasks is challenging, and different approaches are often required for the analysis of different types of images. To facilitate image-based screening studies, a number of bioimage informatics software packages have been developed and are publicly available [9]. This chapter provides an overview of the bioimage informatics approaches in image-based studies for drug and target discovery to help readers, including those without bioimage informatics expertise, understand the capabilities, approaches, and tools of bioimage informatics and apply them to advance their own studies. The remainder of this chapter is organized as follows. Section 2 introduces a number of practical screening applications for discovery of potential drugs and targets. Section 3 describes the challenges and approaches for quantitative image analysis, e.g., object detection, segmentation, and tracking. Section 4 introduces techniques for quantification of segmented objectives, including feature extraction, phenotype classification, and clustering. Section 5 reviews a number of prevalent approaches for profiling drug effects based on the quantitative phenotypic data. Section 6 lists major, publicly available software packages of bioimage informatics analysis, and finally, a brief summary is provided in Section 7.

## 2. Example Image-based Studies for Drug and Target Discovery

There are a variety of image-based studies for discovery of drugs, targets, and mechanisms of biological processes. A good starting point for learning about bioimage informatics approaches is to study practical image-based studies, and a number of examples are summarized below.

### 2.1 Multicolor Cell Imaging-based Studies for Drug and Target Discovery

**Figure 2:**
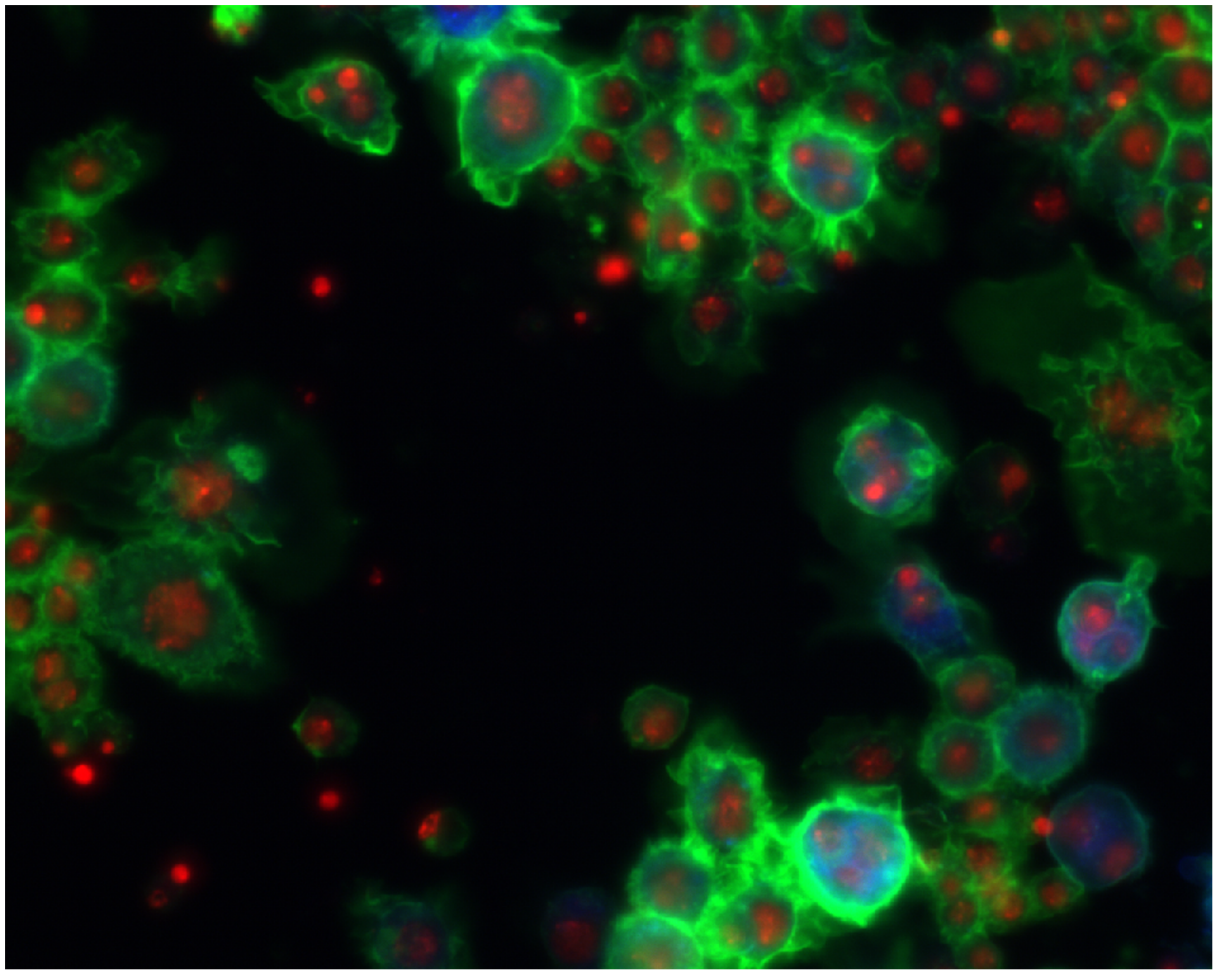
A representative image of *Drosophila* Kc167 cells treated with RNAi. The red, green, and blue colors are the DNA, F-actin, and α-tubulin channels.

Fixed cell images with multiple fluorescent markers have been widely used for drug and target screening in scientific research. For example, the effects of hundreds of compounds were profiled for phenotypic changes using multicolor cell images in [10]–12. Hundreds of quantitative features were extracted to indicate the phenotypic changes caused by these compounds, and then computational approaches were proposed to identify the effective compounds, categorize them, characterize their dose-dependent response, and suggest novel targets and mechanisms for these compounds [10]–[12]. Moreover, phenotypic heterogeneity was investigated by using a subpopulation based approach to characterize drug effects in [13], and distinguish cell populations with distinct drug sensitivities in [14]. Also in [15], [16], the phenotypic changes of proteins inside individual Drosophila Kc167 cells treated with RNAi libraries were investigated by using high resolution fluorescent microscopy, and bioimage informatics analysis was applied to quantify these images to identify genes regulating the phenotypic changes of interest. Figure 2 shows an image of *Drosophila* Kc167 cells, which were treated with RNAi and stained to visualize the nuclear DNA (red), F-actin (green), and α-tubulin (blue). Freely available software packages, such as CellProfiler [17], Fiji [18], Icy [19], GCellIQ [20], and PhenoRipper [21] can be used for the multicolor cell image analysis.

### 2.2 Live-cell Imaging-based Studies for Cell Cycle and Migration Regulator Discovery

**Figure 3:**
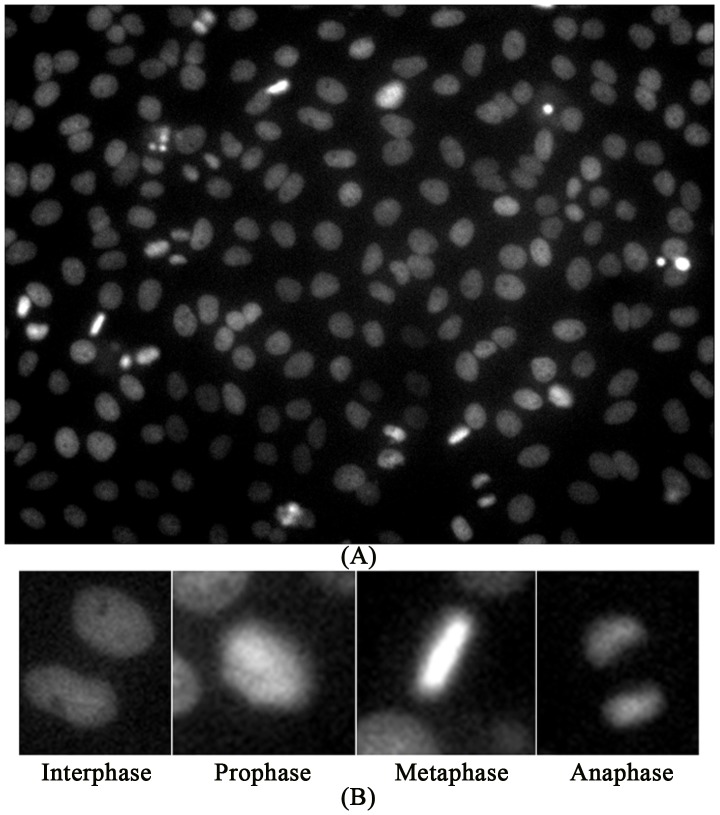
Examples of HeLa cell nuclei and cell cycle phase images. (A) A frame of HeLa cell nuclei time-lapse image sequence; (B) Example images of four cell cycle phases.

Two hallmarks of cancer cells are uncontrolled cell proliferation and migration. These are also good phenotypes for screening drugs and targets that regulate cell cycle progression and cell migration in time-lapse images. For example, out of 22,000 human genes, about 600 were identified as related to mitosis by using live cell (time-lapse) imaging and RNAi treatment in the MitoCheck project (www.mitocheck.org) [22], [23]. The project is now being expanded to study how these identified genes work together to regulate cell mitosis, in which mistakes can lead to cancer, in the MitoSys (systems biology of mitosis) project (http://www.mitosys.org/). Also, live cell imaging of Hela cells was used to discover drugs and compounds that regulate cell mitosis in [24], [25]. Moreover, the time-lapse images of live cells were used to study the dynamic behaviors of stem cells in [26], [27] and predict cell fates of neural progenitor cells using their dynamic behaviors in [28]. Figure 3 shows a single frame of live HeLa cell images and the images of four cell cycle phases: interphase, prophase, metaphase, and anaphase [25]. The publicly available software packages for time-lapse image analysis include, for example, the plugins of CellProfiler [17], Fiji [18], BioimageXD [29], Icy [19], CellCognition [23], DCellIQ [30], and TLM-Tracker [31].

### 2.3 Neuron Imaging-based Studies for Neurodegenerative Disease Drug and Target Discovery

**Figure 4:**
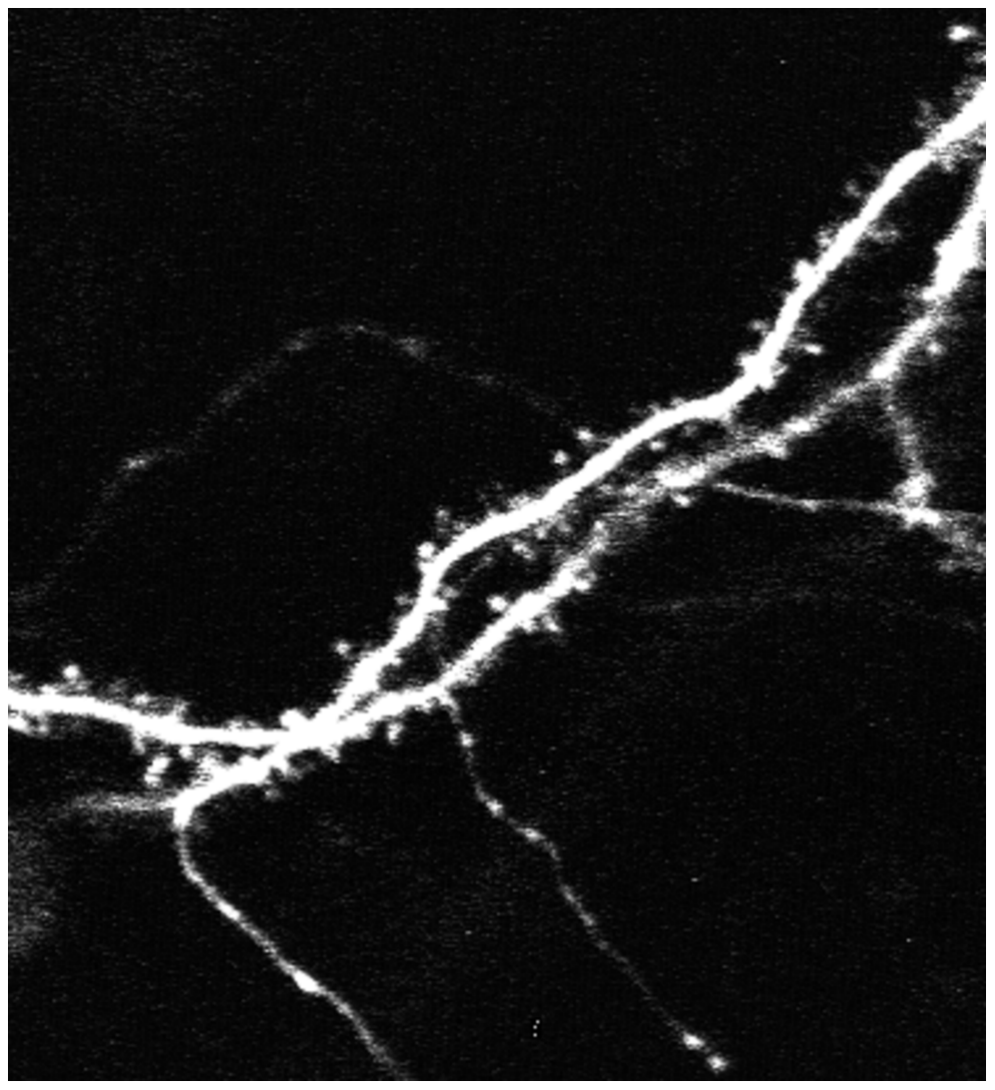
A representative 2D neuron images. The bright spots near the backbones of neurons are the dendritic spines.

**Figure 5:**
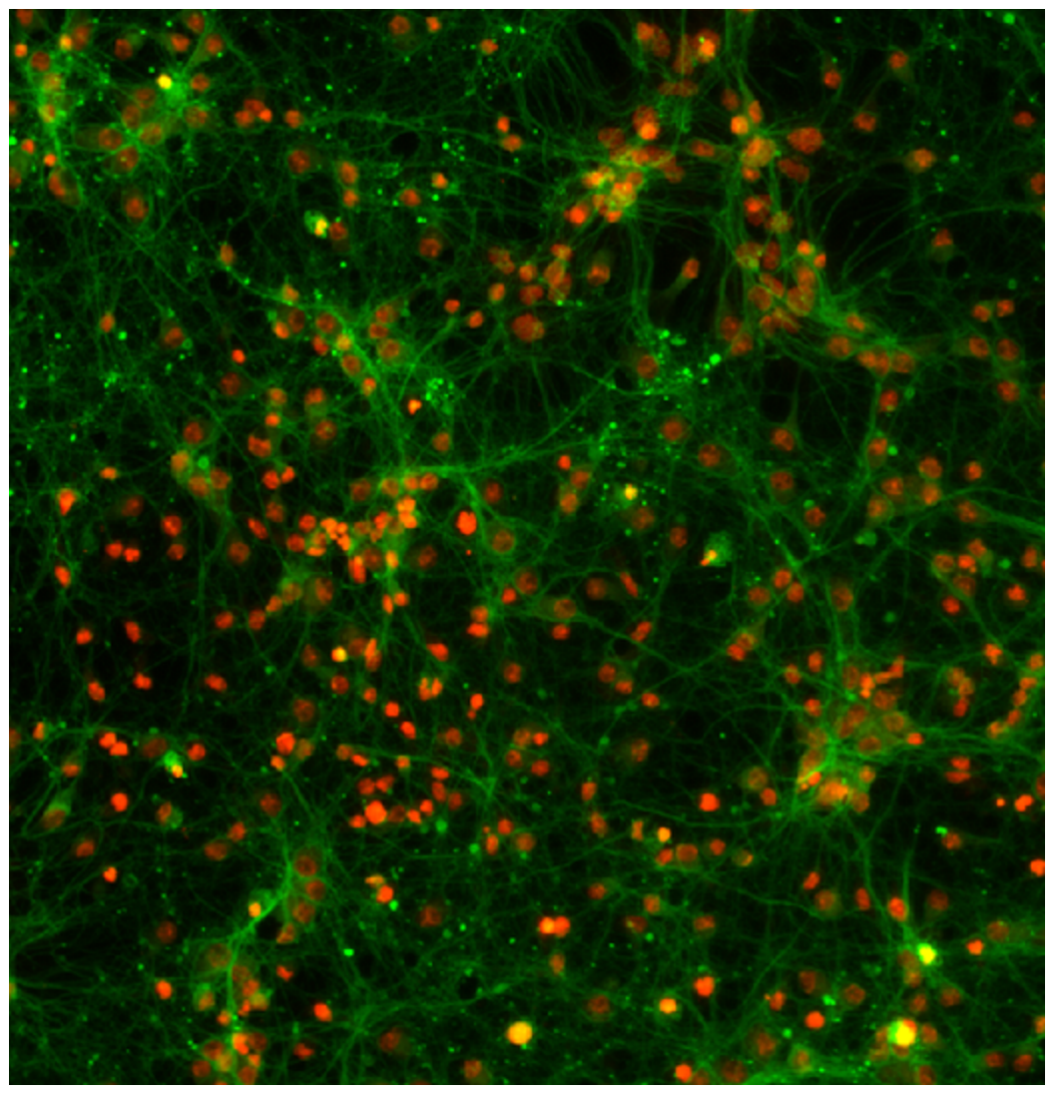
A representative image of neurites. Red indicates nuclei and green represents neurites.

Neuronal morphology is illustrative of neuronal function and can be instructive toward the dysfunctions seen in neurodegenerative diseases, such as Alzheimer's and Parkinson's disease [32], [33]. For example, the 3D neuron synaptic morphological and structural changes were investigated by using super-resolution microscopy, e.g., STED microscopy, to study brain functions and disorders under different stimulations [34]–[36]. Also other advanced optical techniques were proposed in [37], [38] to image and reconstruct the 3D structure of live neurons. Figure 4 shows an example of 2D neuron image used in [39]. In [40], neuronal degeneration was mimicked by treating mice with different dosages of Aβ peptide, which may cause the loss of neuritis, and drugs that rescue the loss of neurites were identified as candidates for AD therapy. Figure 5 shows an example of neurites and nuclei images acquired in [40]. To quantitatively analyze neuron images, a number of publicly available software packages have been developed, for example, NeurphologyJ [41], NeuronJ [42], NeuriteTracer (Fiji plugin) [43], NeuriteIQ [44], NeuronMetrics [45], NeuronStudio [46], [47], NeuronJ [42], NeuronIQ [39], [48], and Vaa3D [49], [50]. A review of software packages for neuron image analysis was also reported in [51].

### 2.4 *Caenorhabditis elegans* Imaging-based Studies for Drug and Target Discovery

*Caenorhabditis elegans (C. elegans)* is a common animal model for drug and target discovery. Consisting of only hundreds of cells, it is an excellent model to study cellular development and organization. For example, the invariant embryonic development of *C. elegans* was recorded by time-lapse imaging, and the embryonic lineages of each cell were then reconstructed by cell tracking to study the functions of genes underpinning the development process [52]–[54]. Moreover, an atlas of *C. elegans*, which quantified the nuclear locations and statistics on their spatial patterns in development, was built based on the confocal image stacks via the software, CellExplorer [55], [56]. In addition, CellProfiler provides an image analysis pipeline for delineating bodies, and quantifying the expression changes of specific proteins, e.g., clec-60 and pharynx, of individual *C. elegans* under different treatments [57].

These examples have demonstrated diverse cellular phenotypes in different image-based studies. To quantify and analyze the complex phenotypic changes of cells and sub-cellular components from large scale image data, bioimage informatics approaches are needed.

## 3. Quantitative Bioimage Analysis

After image acquisition, phenotypic changes need to be quantified for characterizing functions of drugs and targets. Due to the large amounts of images generated, it is not feasible to quantify the images manually. Therefore, automated image analysis is essential for the quantification of phenotypic changes. In general, the challenges of quantitative image analysis include object detection, segmentation, tracking, and visualization. The word ‘object’ in this context means the object captured in the bioimages, e.g., the nucleus and cell. The following sections will introduce techniques used to address these challenges.

### 3.1 Object Detection

Object detection is to detect the locations of individual objects. It is important, especially when the objects cluster together, to facilitate the segmentation task by providing the position and initial boundary information of individual objects. Based on the shape of objects, two categories of object detection techniques are developed: blob structure detection, e.g., particles and cell nuclei, and tube structure detection, e.g., neurons, blood vessels.

**Figure 6:**
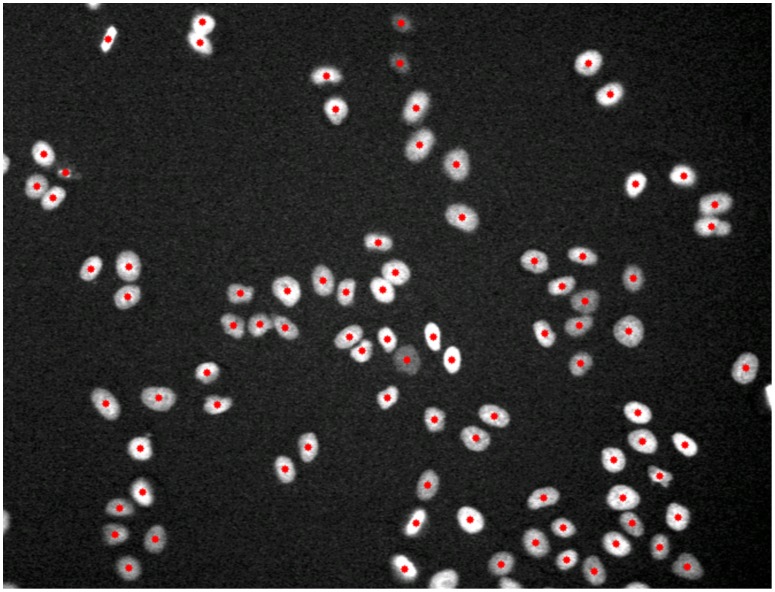
An example of blob-structure (HeLa cell nuclei) detection. The red spots indicate the detected centers of objects.

The shape information of blob objects can be used to detect the centers of objects using distance transformation [58]. The concavity of two touching objects would cause two local maxima in the distance image, such that thresholding or seeded watershed can be employed to the distance image to detect and separate the touching blob objects [59]. The intensity information is also often used for blob detection. Blob objects usually have relatively high intensity in the center, and relatively low intensity in the peripheral regions. For example, the Laplacian-of-Gaussian (LOG) filter is effective [60]–[63] to detect blob objects based on the intensity information. After LOG filtering, local maximum response points often correspond to centers of blob objects, as shown in Figure 6. Moreover, the intensity gradient information is also used for blob detection. For example, in [64] the intensity gradient vectors were smoothed by using the gradient vector flow approach [65] so that the smoothed gradient vectors continuously point to the object centers. Consequently, the blob object centers can be detected by following the gradient vectors [64]. In addition, the boundary points of blob objects with high gradient amplitude can be used to detect their centers, based on the idea of Hough Transform [66]. For example, in [67] an iterative radial voting method was developed to detect such object centers based on the boundary points. In brief, the detected boundary points vote the blob center with oriented kernels iteratively, and the orientation and size of the kernels are updated based on the voting results. Finally, the maximum response points in the voting image are selected as the centers of objects. The advantage of this method is that it can detect the centers of objects with noise appearance [67]. The distance transform and the intensity gradient information also can be combined for the object detection [68]. For other blob objects with complex appearances, the machine learning approaches based on local features [69], [70] can also be used for object detection [71], [72], as in the Fiji (trainable segmentation plugin) [18] and Ilastik [73].

**Figure 7:**
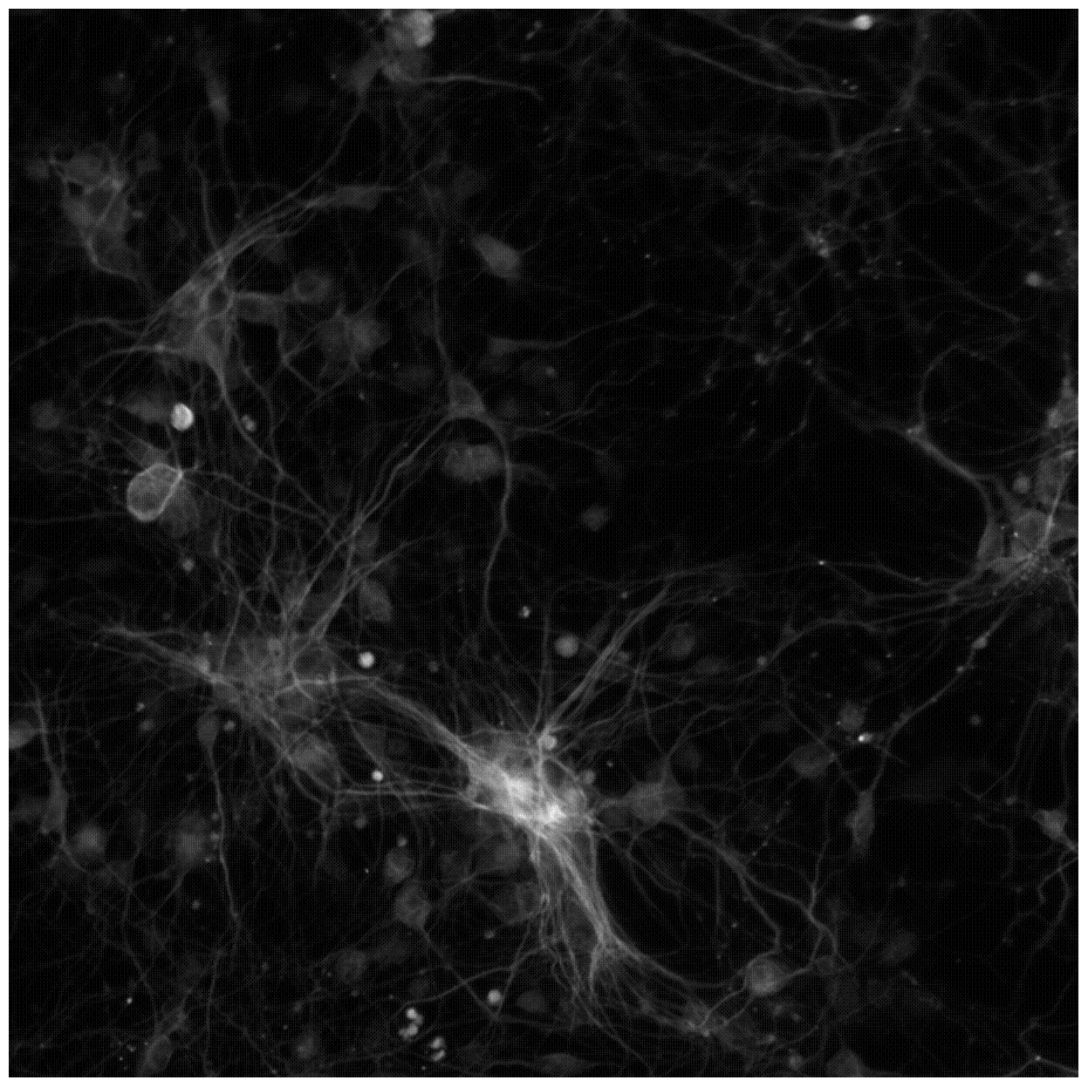
A representative neurite image for centerline detection.

**Figure 8:**
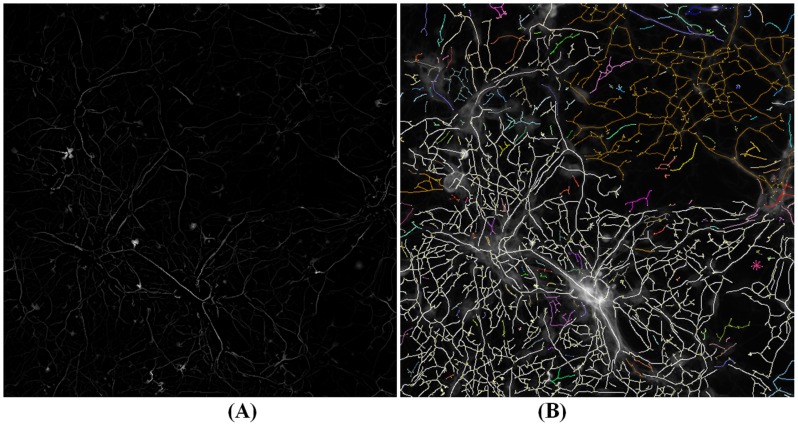
An example of neurite centerline detection. (A) The centerline confidence image obtained by using the local Gaussian derivative features. Higher intensity indicates higher confidence of pixels on the centerlines. (B) The neurite centerline detection result image. Different colors indicate the disconnected branches.

Tubular structure detection is based on the premise that the intensity remains constant in the direction along the tube, and varies dramatically in the direction perpendicular to the tube. To find the local direction of tube center lines, the eigenvector corresponding to the minimum and negative eigenvalue of Hessian matrix was proposed in [44], [74]. Center line points can be characterized by their local geometric attributes, i.e., the first derivative is close to zero and the magnitude of second derivatives is large in a direction perpendicular to tube center line [42], [44], [74]. After the center line point detection, a linking process is needed to connect these center line points into continuous center lines based on their direction and distance. For example, in NeuronJ, Dijkstra's shortest-path was used based on the Gaussian derivative features to detect the neuron's centerline between two given points on the neuron [42]. Figure 7 provides an example of neurite images, and Figure 8 shows the corresponding centerline detection results [44] based on the local Gaussian derivative features. In addition to the approaches based on Gaussian derivatives, there are other tubular structure detection approaches. For example, four sets of kernels (edge detectors) were designed to detect the neuron edges and centerlines [75], and super-ellipsoid modeling was designed to fit the local geometry of blood vessels [76].

Moreover, machine learning-based tubular structure detection is a widely used method. For example, blood vessel detection in retinal images is a representative tubular structure detection task with the supervised learning approaches [77], [78]. In these methods, the local features, e.g., intensity and wavelet features, of an image patch containing a given pixel are calculated, and then a classifier is trained using these local features based on a set of training points [77], [78]. A good survey of blood vessel (tube structure) detection approaches in retinal images was reported in [79]. For more approaches and details of tubular structure detection, readers should refer to the aforementioned neuron image analysis software packages.

In summary, blobs and tubes are the dominating structures in bioimages. The detection results provide the position and initial boundary information for the quantification and segmentation processes. In other words, the segmentation process tries to delineate boundaries of objects starting from the detected centers or centerlines of objects. Without the guidance of detection results, object segmentation would be more challenging.

### 3.2 Object Segmentation

The goal of object segmentation is to delineate boundaries of individual objects of interest in images. Segmentation is the basis for quantifying phenotypic changes. Although a number of image segmentation methods have been reported, this remains an open challenge due to the complexity of morphological appearances of objects. This section introduces a number of widely used segmentation methods.

**Figure 9:**
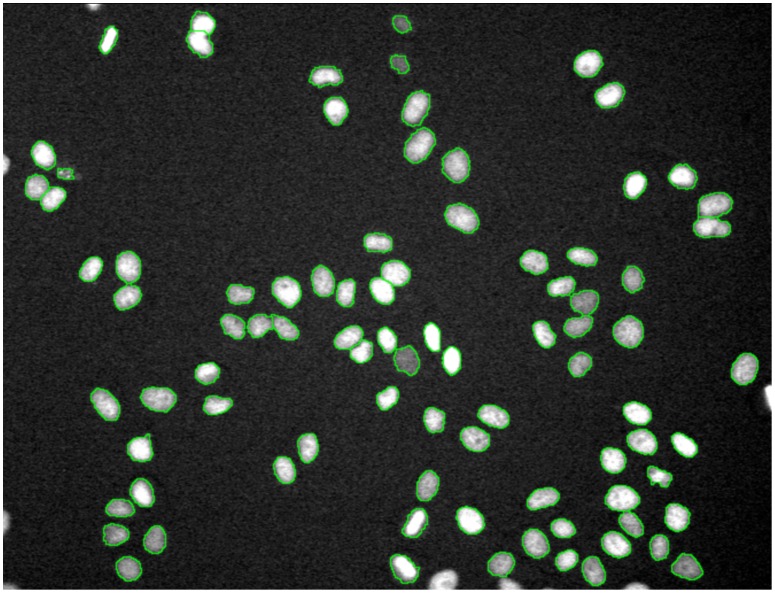
An example of HeLa nuclei segmentation using the seeded watershed algorithm. The green contours are the boundaries of nuclei.

Threshold segmentation [80] is the simplest method: 
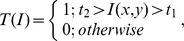
 where *I*(*x*,*y*) is the image, and *t_1_* and *t_2_* are the intensity thresholds. As an extension of the thresholding method, Fuzzy-C-Means [81] can be used to separate images into more regions based on intensity information. These methods could divide the image into objects and background, but fail to separate the object clumps (i.e., multiple objects touching together). Watershed segmentation and its derivatives are widely used segmentation methods. They build object boundaries between objects on the pixels with local maximum intensity, which act like dams to avoid flooding from different basins (object regions) [82]. To avoid the over-segmentation problem of the watershed approach, the marker-controlled watershed (or seeded watershed) approach, in which the floods are from the ‘marker’ or ‘seed’ points (the object detection results), was proposed [68], [83]–[85]. Figure 9 illustrates the segmentation result of HeLa cell nuclei using the seeded watershed method based on the cell detection results.

**Figure 10:**
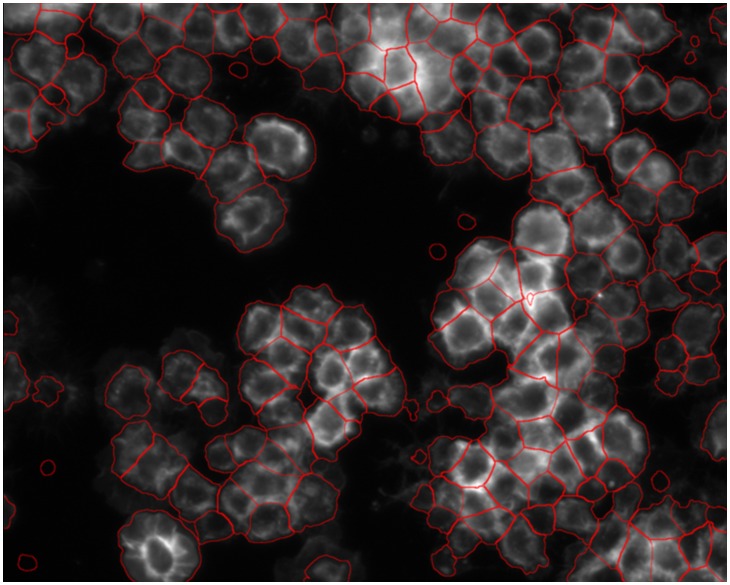
An example of segmentation of *Drosophila* cell images using the level set approach.

Active contour models are another set of widely used segmentation methods [86]–[90]. Generally, there are two kinds of active contour models: boundary-driven and region-competition models. In the boundary-driven model, the contours' (boundaries of objects) evolution is determined by the local gradient. In other words, the boundary fronts move toward the outside (or inside) quickly in the regions with low intensity variation (gradient), and slowly in the regions with high gradient (where the boundaries are). When great intensity variation appears inside cells, or the boundary is weak, this method often fails [91]. Instead of using gradient information, the region-competition model makes use of the intensity similarity information to separate the image into regions with similar intensity. Region competition-based active contour models could solve the weak boundary problem; however, they require that the intensity of touching objects is separable [87]. To implement these active contour models, level set representation is widely used [92]. Level set is an *n+1* dimensional function that can easily represent any *n* dimensional shape without parameters. The inside regions of objects are indicated by using positive levels, and outside regions are represented using negative levels. For this implementation, the initial boundary (zero level) is required, and the signed distance function is often used to initialize the level set function [92], [93]. To evolve the level set functions (grow the boundaries of objects), the following two equations are classical models. The first equation is often called geodesic active contour (GAC) [86], and the second one is often named the Chan and Vese active contour (CV) [87].
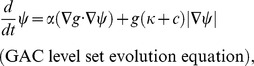


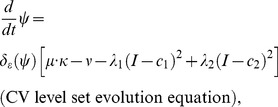
where 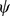 denotes the level-set function, and *g* indicates the gradient function, 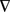 is the gradient operator, *c*, *c_1_*, and *c_2_* are constant variables. 
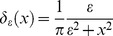
 is an approximation of the Dirac function to indicate the boundary bands), which is the derivative function of Heaviside function denoting inside/outside regions of objects: 
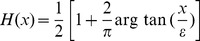
, and the curvature term, 
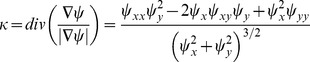
 indicates the local smoothness of boundaries, and ‘*div*’ is the divergence operation. Figure 10 demonstrates the segmentation result using GAC level set approach. An additional segmentation method, Voronoi segmentation [94], first defines the centers of objects and then constructs the boundaries between two objects on the pixels, from which the distances are the same to the two centers. In CellProfiler, the Voronoi segmentation method was extended by considering the local intensity variations in the distance metric to achieve better segmentation results [95]. This method is fast and generates level set comparable results. Graph cut segmentation method views the image as a graph, in which each pixel is a vertex and adjacent pixels are connected [63], [96], [97]. It ‘cuts’ the graph into several small graphs from the regions where adjacent pixels have the most different properties, e.g., intensity.

Different from the aforementioned segmentation approaches, local feature and machine learning-based segmentation approaches are implemented, for example, in Fiji (trainable segmentation plugin) [18] and Ilastik [73]. Users can interactively select the training sample pixels/voxels or small image patches conveniently, and then classifiers are automatically trained based on the features of the training pixels or voxels (or patches) to predict the classes, e.g., cells or background, of the pixels or voxels (or patches) in a new image. The image patches could be a circle or square neighbor regions of a given point, and also could be regions (superpixel) obtained by the clustering analysis. For example, Simple Linear Iterative Clustering (SLIC) made use of the intensity and coordinate information of pixels to separate the image into uniformly sized and biologically meaningful regions [98], [99], and then the machine learning approaches were used to identify the regions of interest, e.g., boundary superpixels, for object segmentation [99].

### 3.3 Object Tracking

**Figure 11:**
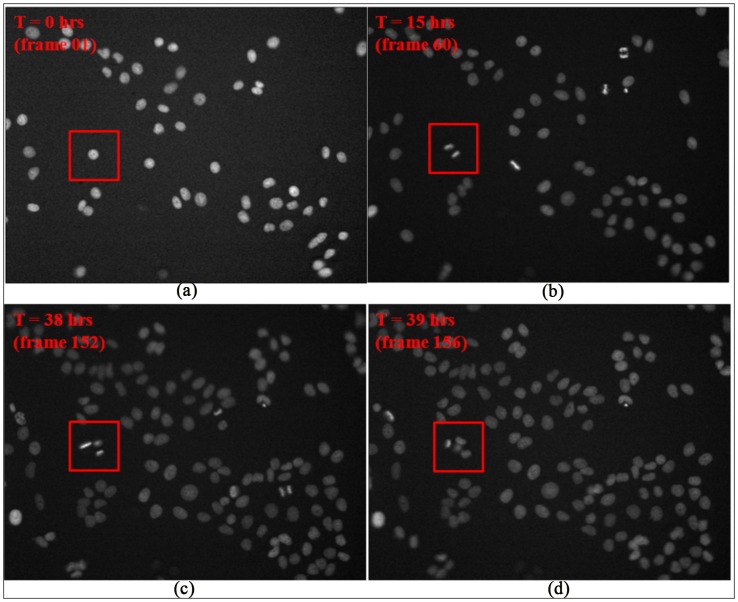
Time-lapse images indicating cell cycle progression. The cell in the red square in the first frame (A) divided into two cells in frame 60 (B). The descendent cells divided again in frame 152 and 156 respectively as shown in the red squares in (C) and (D).

**Figure 12:**
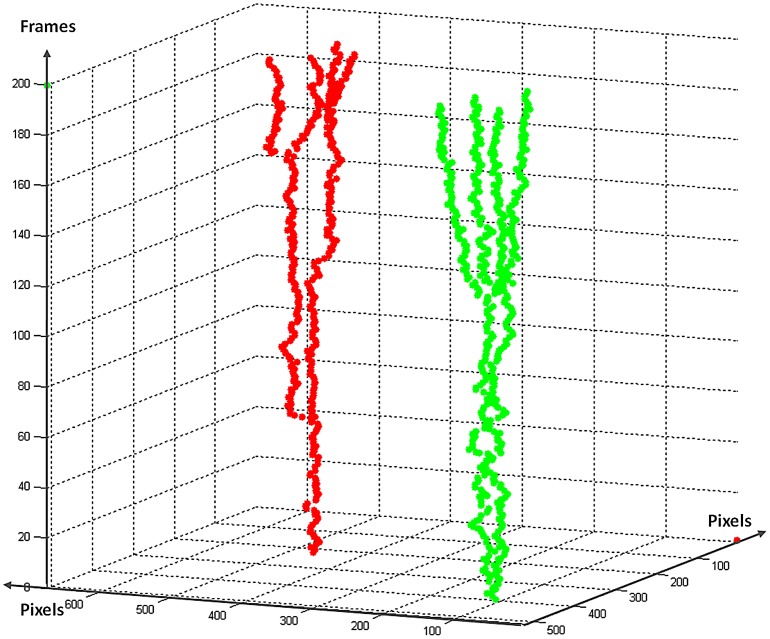
Examples of cell migration trajectories. Different colors represent different trajectories.

**Figure 13:**
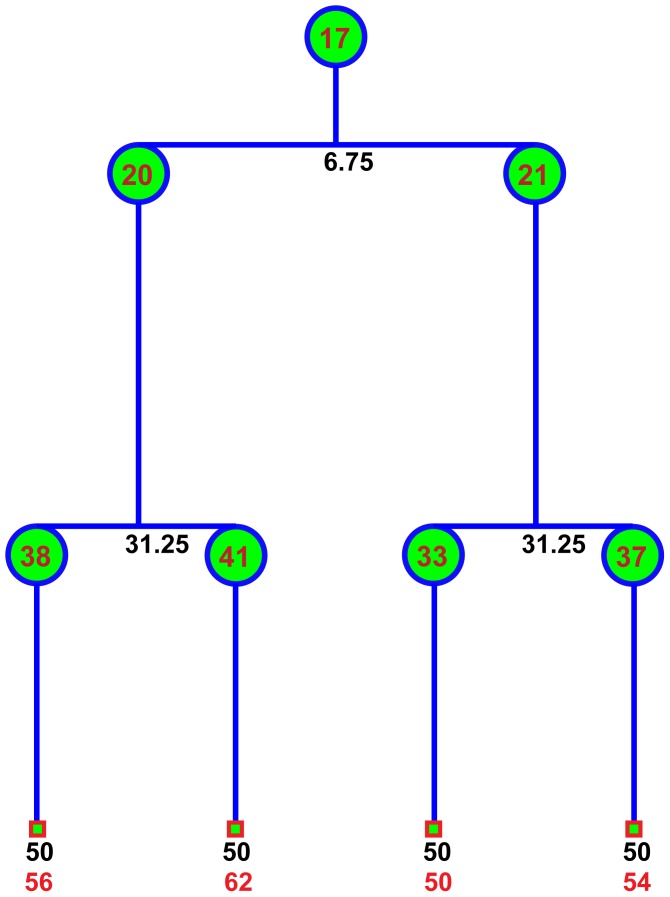
Examples of cell lineages constructed by the tracking algorithm. The black numbers are the time of cell division (hours). The bottom red numbers indicate the number of traces, and the numbers inside circles are the labels of cells in that frame.

To study the dynamic behaviors and phenotypic changes of objects over time (e.g., cell cycle progression and migration), object tracking using time lapse image sequences is necessary. Figure 11 shows a Hela cell's division process in four frames at different time points, and Figures 12 and 13 show the examples of cell migration trajectories and cell lineages reconstructed from the time-lapse images of Hela cells [30]. Object tracking is a challenging task due to the complex dynamic behaviors of objects over time. In general, cell tracking approaches can be classified into three categories: model evolution-based tracking, spatial-temporal volume segmentation-based tracking, and segmentation-based tracking.

In the model evolution based tracking approaches, cells or nuclei are initially detected and segmented in the first frame, and then their boundaries and positions evolve frame by frame. Some tracking techniques in this category are mean-shift [100] and parametric active contours [88], [101]. However, neither mean-shift nor parametric active contours can cope well with cell division and nuclei clusters. Though the level set method enables topological change, e.g., cell division, it also allows the fusion of overlapping cells. Extending these methods to cope with these tracking challenges is nontrivial and increases computation time [90], [102]–[104]. For example, the coupled geometric active contours model was proposed to prevent object fusion by representing each object with an independent level set in [105], and this was further extended to the 3D cell tracking in [90]. The other approach explicitly blocking the cell merging is to introduce the topology constraints, i.e., labeling objects regions with different numbers or colors. For example, the region labeling map was employed in [27], [106] to deal with the cell merging, and planar graph–vertex coloring was employed to separate the neighboring contours. From that four separate level set functions could easily deal with cell merging [107] based on the four-color theorem [108], [109]. For the spatial-temporal volume segmentation based tracking, 2D image sequences were viewed as 3D volume data (2D spatial+temporal), and the shape and size constrained level set segmentation approaches were applied to segment the traces of objects, and reconstruct the cell lineage in [110]–[112].

For detection and segmentation-based tracking, objects are first detected and segmented, and then these objects are associated between two consecutive frames, based on their morphology, position, and motion [30], [113]–[115]. The tracking approaches are usually done fast, but their accuracy is closely related to detection and segmentation results, similarity measurements, and association strategies. The cell center position, shape, intensity, migration distance, and spatial context information were used as similarity measurements in [113], [115]. For the association approaches, the overlap region and distance based method was employed in [114], in which objects in the current frame were associated with the nearest objects in the next frame. Then the false matches, e.g., many-to-one or one-to-many, were further corrected through the post processing. Different from the individual object association above, all segmented objects were simultaneously associated by using the integer programming optimization in [113], [116]: 
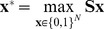
, *s.t.* 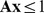
_,_ where 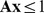 restricts that one object can be associated to one object at most, **A** is an (*m+n*)×*N* matrix, and the first *m* rows correspond to *m* objects in frame *t*, and the last *n* rows denote objects in frame *t+1*. *N* is the number of all possible associations among objects in frame *t* and frame *t+1*. **S** is a 1×*N* similarity matrix, and 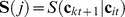. For the unmatched cells, e.g., the new born or new entered cells, a linking process is usually needed to link them to the parent cells or as a new trajectory. This optimal matching strategy was also used to link the object trajectory segments in [27] to link the broken or newly appearing trajectories.

As an alternative to frame-by-frame association strategies, Bayesian filters, e.g., Particle filter and Interacting Multiple Model (IMM) filters [117], [118], are also used for object tracking. The goal of these filters is to recursively estimate a model of object migration in an image sequence. Generally, in the Bayesian methods, a state vector, **x**_t_, is defined to indicate the characters of objects, e.g., position, velocity, and intensity. Then, two models are defined based on the state vector. The first is the state evolution model, **x**_t_ = f_t_ (**x**_t−1_)+ε_t_, where f_t_ is the state evolution function at time point, *t*, and ε_t_ is a noise, e.g., Gaussian noise, which describes the evolution of the state. The other is the observation model, **z**_t_ = h_t_ (**x**_t−1_)+η_t_, where h_t_ is the map function, and η_t_ is the noise, which maps the state vector into observations that are measurable in the image. Based on the two models and Bayes' rule, the posterior density of the object state is estimated as follows: 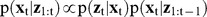, and 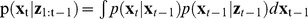 where the p(**z**_t_ |**x**_t_) is defined based on the observation model, and the 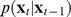 is defined based on the state evolution model. The basic principle of particle filter is to approximate the posterior density by a set of samples (particles) being stochastically drawn, and it had been employed for object tracking in fluorescent images in [119]–[121]. In some biological studies, the motion dynamics of objects are complex. Therefore, one motion model might not be able to describe object motion dynamics well. The IMM filter is employed to incorporate multiple motion models, and the motion model of objects can be transitioned from one to another in the next frame with certain probabilities. For example, the IMM filter with three motion models, i.e., random walk, first-order, and second-order linear extrapolation, was used for 3D object tracking in [118], and for 2D cell tracking in [27].

### 3.4 Image Visualization

Most of the aforementioned software packages provide functions to visualize 2D images and the analysis results. However, for higher dimensional images, e.g., 3D, 4D (including time), and 5D (including multiple color channels), visualization is challenging. Fiji [18], Icy [19], and BioimageXD [29], for example, are the widely used bioimage analysis and visualization software packages for higher dimensional images. In addition, NeuronStudio [46], [47] is a software package tailored for neuron image analysis and visualization. Farsight [122] and vaa3D [123] are also developed for analysis and visualization of 3D, 4D, and 5D microscopy images. For developing customized visualization tools, the Visualization Toolkit (VTK) is a favorite choice (http://www.vtk.org/) as it is open source and developed specifically for 3D visualization. ParaView (http://www.paraview.org/) and ITK-SNAP (http://www.itksnap.org/) are the popular Insight Toolkit (ITK) (http://www.itk.org/) and VTK based 3D image analysis and visualization software packages.

This section has introduced a number of major methods for object detection, segmentation, tracking, and visualization in bioimage analysis. These analyses are essential and provide a basis for the following quantification of morphological changes.

## 4. Numerical Features and Morphological Phenotypes

### 4.1 Numerical Features

To quantitatively measure the phenotypic changes of segmented objects, a set of descriptive numerical features are needed. For example, four categories of quantitative features, measuring morphological appearances of segmented objects, are widely used in imaging informatics studies for object classification and identification, i.e., wavelets features [124], [125], geometry features [126], Zernike moment features [127], and Haralick texture features [128]. In brief, Discrete Wavelet Transformation (DWT) features characterize images in both scale and frequency domains. Two important DWT feature sets are the Gabor wavelet [129] and the Cohen–Daubechies–Feauveau wavelet (CDF9/7) [130] features. Geometry features describe the shape and texture features of the individual cells, e.g., the maximum value, mean value, and standard deviation of the intensity, the lengths of the longest axis, the shortest axis, and their ratio, the area of the cell, the perimeter, the compactness of the cell (compactness = perimeter∧2/4π*area), the area of the minimum convex image, and the roughness (*area of cell/area of convex shape*). The calculation of Zernike moments features was introduced in [131]. First, the center of mass of the cell image was calculated, then the average radius for each cell was computed, and the pixel p(x, y) of the cell image was mapped to a unit circle to obtain the projected pixel as p(x′, y′). Then Zernike moment features were calculated based on the projected image I(x′, y′). The Haralick texture features are extracted from the gray-level spatial-dependence matrices, including the angular second moment, contrast, correlation, sum of the squares, inverse difference moment, sum of the average, sum of the variance, sum of entropy, entropy, difference of the variance, difference of entropy, information measures of correlation, and maximal correlation coefficient [132]. More descriptions and calculation programs about these Subcellular Location Features (SLF) and SLF-based machine learning approaches for image classification can be found at: http://murphylab.web.cmu.edu/services/SLF/features.html.

### 4.2 Phenotype Identification

Although these numerical features are informative to describe the phenotypic changes, it can be difficult to understand these changes in terms of visual and understandable phenotypic changes. For example, the increase or decrease of cell size can be understood; however, it is not clear what the physical meaning of the increase or decrease is for certain wavelet features. Therefore, transforming the numerical features into biologically meaningful features (phenotypes) is important. This section introduces a number of widely used phenotype identification approaches.

#### 4.2.1. Cell cycle phase identification

**Figure 14:**
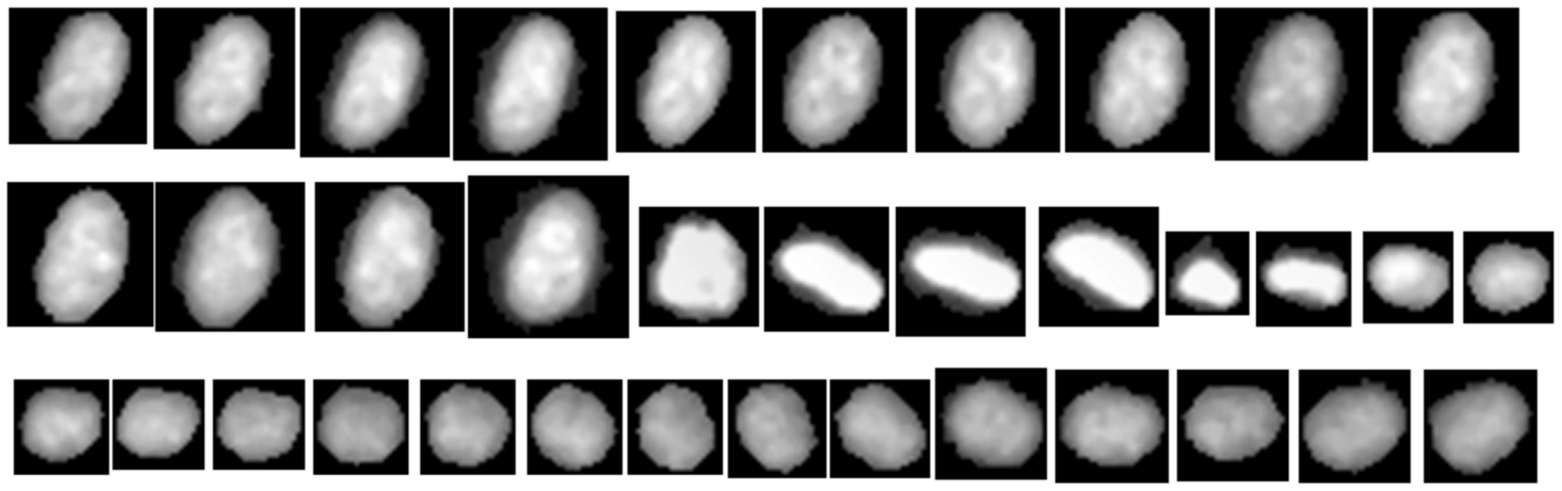
A segment of cell cycle procession sequence. Four cell cycle phases, interphase, prophase, metaphase, and anaphase, appear in order.

In cell cycle studies, drug and target effects are indicated by the dwelling time of cell cycle phases, e.g., interphase, prophase, metaphase and anaphase. Additional cell cycle phases, e.g., Prometa-, Ana 1-, Ana 2-, and Telo- phases, were also investigated in [133] and [23], [134]. After object segmentation and tracking, cell motion traces can be extracted, as shown in Figure 14, and then the automated cell cycle phase identification is needed to calculate the dwelling time of individual cells on different phases.

**Figure 15:**
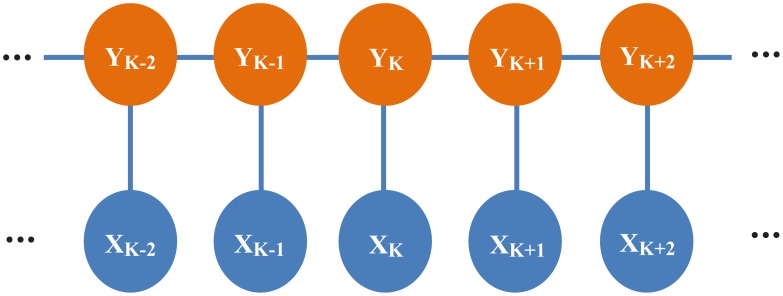
The graphical representation of cell cycle phase identification.

Cell cycle phase identification can be viewed as a pattern classification problem. The aforementioned numerical features, and a number of classifiers can be used to identify the corresponding phases of individual segmented cells, e.g., support vector machine (SVM) [115], [133], [135], K-nearest neighbors (KNN), and naïve Bayesian classifiers [114]. However, the classification accuracy is often poor for cell cycle phases appearing for a short time, e.g., prophase and metaphase, due to the unbalance of sample size compared to interphase, and the segmentation bias. Fortunately, the cell cycle phase transition rules, e.g., from interphase to prophase, and from prophase to metaphase, can be used to reduce identification errors. Thus, a set of cell cycle phase identification approaches based on the cell tracking results were proposed to achieve high identification accuracy. This problem is often formulized as follows, and as shown in Figure 15. Let **x** = (*x*_1_, *x*_2_, …, *x*_T_) denote a cell image sequence of length *T*. Each cell image is represented by a numerical feature vector 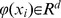 (using the aforementioned numerical features). Let **y** = (*y*_1_, *y*_2_, …, *y*_T_) represent the corresponding cell cycle phase sequence that needs to be predicted. Based on the cell cycle progression rules, for example, the variation of nuclei size and intensity were used as an index to identify the mitosis phases of cells in [25], and Hidden Markov Modeling (HMM) was used to identify the cell cycle phases in CellCognition [23]. In brief, the transition possibility from one phase to the other was learned from the training data of cell cycle progressions, which could improve the accuracy of cell cycle phase identification. As an extension of HMM, Temporally Constrained Combinatorial Clustering (TC3), which is an unsupervised learning approach for cell cycle phase identification, was designed and combined with Gaussian Mixture Model (GMM) and HMM to achieve robust and accurate cell cycle identification results in [134]. Also, in [133] Finite State Machine (FSM) was employed to check the phase transition consistency and make corrections to the error cell cycle phases predicted by using SVM classifier [115]. Moreover, the cell cycle phases could be identified during the segmentation and linking process in the spatiotemporal volumetric segmentation-based tracking methods [110]–[112].

#### 4.2.2 User defined phenotype, identification, and classification

**Figure 16:**
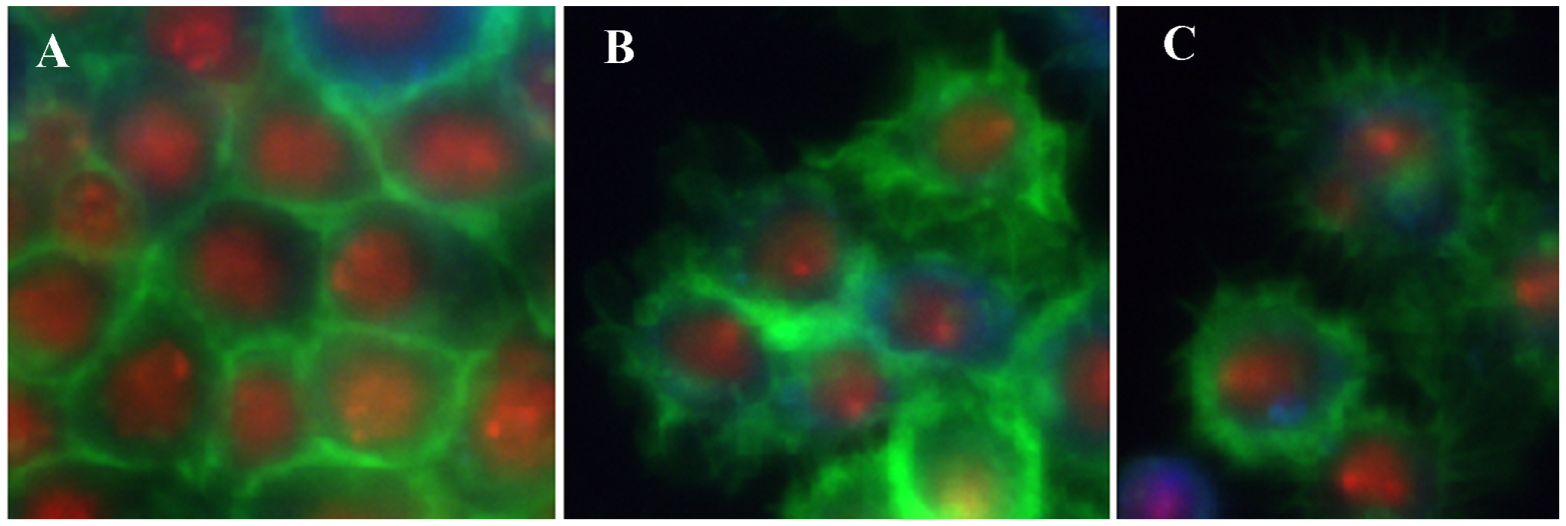
A representative image of *Drosophila* cells with three phenotypes: (A) Normal, (B) Ruffling and (C) Spiky phenotypes.

In certain image-based studies, cells may not have an intrinsic phenotype, e.g., cell cycle phases, but may exhibit unpredicted and novel phenotypes caused by experimental perturbations, e.g., drugs or RNAi treatments. These phenotypes are often defined by well-trained biologists to characterize drug and target effects [16]. Figure 16 shows images of *Drosophila* cells with three defined phenotypes: Normal, Ruffling and Spiky [136].

In large scale screening studies, however, it is subjective and time-consuming for biologists to uncover novel phenotypes from millions of cells. Thus, automated discovery of novel phenotypes is important. For example, an automated phenotype discovery method was proposed in [20]. In brief, a GMM was constructed first for the existing phenotypes. Then the quantitative cellular data from new cellular images were combined with samples generated from the GMM, and the cluster number of the combined data was estimated using gap statistics [137]. Then, clustering analysis was performed on the combined data set, in which some of the cells from the new cellular images were merged into the existing phenotypes, and the clusters that could not be merged by any existing phenotype classes were considered as new phenotype candidates. After the phenotypes are defined, classifiers can be built conveniently based on the training data and the numerical features for classifying cells into one of the predefined phenotypes. However, it is tedious to manually collect enough training samples of the rare and unusual phenotypes. To solve this challenge, an iterative machine learning based approach was proposed in [138]. First, a tentative rule (classifier) was determined based on a few samples of a given phenotype, and then the classifier presented users a set of cells that were classified into the phenotype based on the tentative rule. Users would then manually correct the classification errors, and the corrections are used to refine the rule. This method could collect plenty of training samples after several rounds of error correction and rule refinement [138].

This section introduced numerical feature extraction, phenotype identification, and classification. These analyses provide quantitative phenotypic change data for identifying candidate targets and drug hits that cause desirable phenotypic changes. The following section will describe approaches to analyze the quantitative phenotypic profile data for drug and target identification.

## 5. Multidimensional Profiling Analysis

The aim of profiling analysis is to characterize the functions of drugs and targets, divide them into groups with similar phenotypic changes, and identify the candidates causing desired phenotypic changes. To help analyze and organize these multidimensional phenotypic profile data, some publicly available software packages have been designed, for example, CellProfiler Analyst (http://www.cellprofiler.org/) and PhenoRipper (http://www.phenoripper.org). In addition, KNIME (http://www.knime.org/) is a publicly available pipeline and workflow system to help organize different data flows. It also provides connections to bioimage analysis software packages, e.g., Fiji [18] and CellProfiler [9], and enables users to conveniently build specific data analysis pipelines in KNIME. This section describes some prevalent approaches in analyzing quantitative phenotypic profile data.

### 5.1 Clustering Analysis

Clustering analysis is to divide experimental perturbations, e.g., drugs, RNAis, into groups that have similar phenotypic changes. As clustering analysis approaches, e.g., Hierarchical Clustering [139] and Consensus Clustering [140], are well established, their technical details will not be discussed here. In addition to the aforementioned software, Cluster 3.0 (http://www.falw.vu/~huik/cluster.htm) and Java TreeView (http://jtreeview.sourceforge.net/) are two additional easy-to-use clustering analysis software packages available in public domain.

### 5.2 SVM-based Multivariate Profiling Analysis

**Figure 17:**
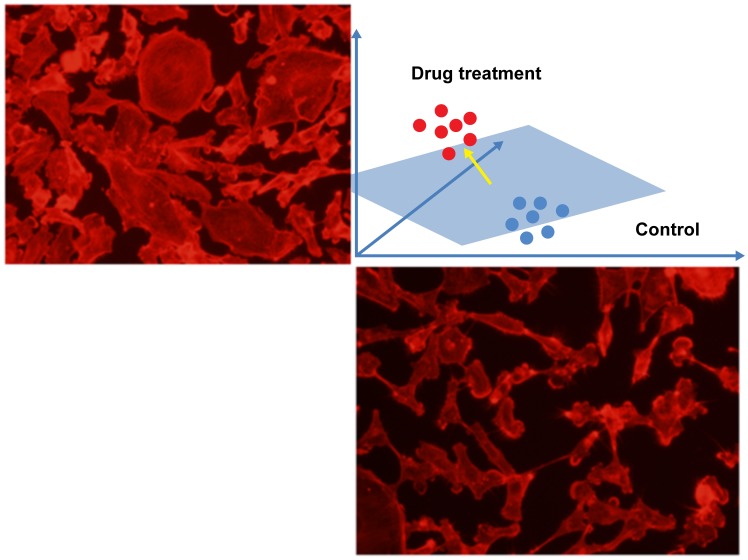
An illustration of drug profiling using the normal vector of hyperplane of SVM. The red and blue spots indicate the spatial distribution of cells in the numeric feature space. The yellow arrow represents the normal vector of the hyperplane (the blue plane). The top left and bottom right (MB231 cell) images are from drug treated and control conditions respectively.

SVM classifier was employed for analyzing the multivariate drug profiles in [141]. To measure the phenotypic change caused by drug treatments, the cell populations harvested from the drug-treated wells were compared with cells collected from the control wells (no drug treatment). The difference between the control and drug treatment was indicated by two factors that are the outputs of the SVM classifier. One is the accuracy of classification, which indicates the magnitude of the drug effect. The other is the normal vector (d-profile) of the hyperplane separating the two cell populations, which indicates the phenotypic changes caused by the drug. Figure 17 illustrates the idea; the yellow arrow is the d-profile indicating the direction of drug effects in the phenotypic feature space. Drugs with similar d-profiles were found to have the same functional targets, and thus it could be used to predict functions of new drugs or compounds.

### 5.3 Factor-based Multidimensional Profiling Analysis

In the set of numerical features, some are highly correlated within groups but poorly correlated with features in other groups. One possible explanation is that the features in one group measure a common biological process, such as increase or decrease of nuclei size. The challenge using these numerical features directly is that biological meanings of certain phenotypic features are often vague. It is thus difficult to explain the phenotypic changes represented by these numerical features as aforementioned. To remove the redundant features and make the biological meanings of numerical features explicitly clear, factor analysis was employed in [12]. The basic principle of factor analysis is to determine the independent common ‘traits’ (factors). Mathematically it is formulated by the following equation.
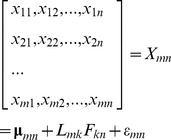
where 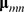 is the mean value of each row, *F_kn_* denotes the *k* factor, and the *L_mk_* is the loading matrix, which is the coordinates of the *n* samples in the new k-dimensional space. In other words, *k* factors are independent and are the underlying biological processes that regulate the phenotypic changes. For example, six factors representing nuclei size, DNA replication, chromosome condensation, nuclei morphology, Edu texture, and nuclei ellipticity, were obtained through factor analysis in [12].

### 5.4 Subpopulation-based Heterogeneity Profiling Analysis

In image-based screening studies, heterogeneous phenotypes often appeared within a cell population, as shown in Figures 2 and 16, which indicated that individual cells responded to perturbations differently [142]. However, the heterogeneity information was ignored in most screening studies. To better make use of the heterogeneous phenotypic responses, a subpopulation based approach was proposed to study the phenotypic heterogeneity for characterizing drug effects in [13], and distinguishing cell populations with distinct drug sensitivities in [14]. The basic principle of the subpopulation based method is to characterize the phenotypic heterogeneity with a mixture of phenotypically distinct subpopulations. This idea was implemented by fitting a GMM in the numerical space, and each model component of the GMM represents a distinct subpopulation. To profile the effects of perturbations, cells collected from perturbation conditions were first classified into one of the subpopulations, and then the portions of cells belonging to each subpopulation were calculated as features to further characterize the effects of perturbations. For more details, please refer to [13], [14].

## 6. Publicly Available Bioimage Informatics Software Packages

A number of commercial bioimage informatics software tools e.g., GE-InCellAnalyzer [143], Cellomics [144], Cellumen [145], MetaXpress [146], BD Pathway [147] have been developed and are widely used in pharmaceutical companies, and academic institutions. In addition to the commercially available software packages, there are a number of publicly available bioimage informatics software packages [9], which provide even more powerful functions with cutting-edge algorithms and screening-specific analysis pipelines. For the convenience of finding these popular software packages, they are listed in Table 1. It is difficult to summarize all of their capabilities and functions because many of them are designed for flexible bioimage analysis with a set of diverse plugins and function modules, e.g., Fiji, CellProfiler, Icy, and BioimageXD. The software selection for specific applications is also non-trivial, and the best way might be to check their websites and online documents. In addition to the bioimage informatics software packages, there are other software packages, including the microscope control software for image acquisition (μManager and ScanImage) and image database software (OME, Bisque and OMERO.searcher). Also, certain cellular image simulation software packages, e.g., CellOrganizer and SimuCell, provide useful insights into the organizations of proteins of interest within individual cells. These software packages represent the prevalent directions of bioimage informatics research, thus their websites and features are worth checking.

**Table 1:** List of publicly available bioimage informatics software packages.

Name	Link	Basic Functions
ImageJ	http://rsb.info.nih.gov/ij/	General image analysis with rich plugins
Fiji (A distribution of ImageJ)	http://fiji.sc/	Bioimage analysis with rich plugins
CellProfiler	http://www.cellprofiler.org/	Bioimage analysis with rich analysis pipelines
CellProfiler Analyst	http://www.cellprofiler.org/	Screening data analysis with machine learning approaches
Icy	http://icy.bioimageanalysis.org/index.php	Bioimage analysis
BioimageXD	http://www.bioimagexd.net/	3D Bioimage analysis and Visualization
PhenoRipper	http://www.phenoripper.org	Bioimage analysis for rapid exploration and interpretation of bioimage data in drug screening
FarSight	http://www.farsight-toolkit.org/wiki/Main_Page	Dynamic Biological Microenvironments from 4D/5D Microscopy Data
Vaa3D	http://penglab.janelia.org/proj/v3d/V3D/About_V3D.html	Bioimage visualization and analysis
Cell Analyzer	http://penglab.janelia.org/proj/cellexplorer/cellexplorer/What_is_Cell_Explorer.html	*C. elegans* image analysis
AceTree and StarryNite	http://starrynite.sourceforge.net/	*C. elegans*' embryo cell tracking and lineage reconstruction
Ilastik	http://www.ilastik.org/	Image classification and segmentation
Image Quantitators (ZFIQ, DCellIQ, GCellIQ, NeuriteIQ, NeuronIQ)	http://www.methodisthealth.com/bbpsoftware	A set of image analysis software packages for cell tracking in time-lapse images, and RNAi cell, neuron, neurite and Zebrafish image analysis
CellCognition	http://cellcognition.org/software/cecoganalyzer	Cell tracking in time-lapse image analysis
TLMTracker	http://www.tlmtracker.tu-bs.de/index.php/Main_Page	Cell tracking in time-lapse image analysis
NeuronJ	http://www.imagescience.org/meijering/software/neuronj/	Neurite Tracing and Quantification
NeurphologyJ	http://life.nctu.edu.tw/~microtubule/neurphologyJ.html	Neuron image analysis
NeuronStudio	http://research.mssm.edu/cnic/tools-ns.html	Neuron image analysis
CellOrganizer	http://cellorganizer.org/	Synthetically model and simulate fluorescent microscopic cell images
SimuCell	http://www.simucell.org	Synthetically model and simulate fluorescent microscopic cell images
PatternUnmixer	http://murphylab.web.cmu.edu/software/PatternUnmixer2.0/	Model fundamental sub-cellular patterns
μManager	http://valelab.ucsf.edu/~MM/MMwiki/	Control of automated microscopes
ScanImage	http://openwiki.janelia.org/wiki/display/ephus/ScanImage%2CEphus%2CandotherDAQsoftware	Control of automated microscopes
OME	http://www.openmicroscopy.org/site	Image Database Software
Bisque	http://www.bioimage.ucsb.edu/bisque	Image Database Software
OMERO.searcher	http://murphylab.web.cmu.edu/software/searcher/	Content-based bioimage search
KNIME	http://www.knime.org/example-workflows	Workflow system for data analytics, reporting and integration

## 7. Summary

With the advances of fluorescent microscopy and robotic handling, image-based screening has been widely used for drug and target discovery by systematically investigating morphological changes within cell populations. The bioimage informatics approaches to automatically detect, quantify, and profile the phenotypic changes caused by various perturbations, e.g., drug compounds and RNAi, are essential to the success of these image-based screening studies. In this chapter, an overview of the current bioimage informatics approaches for systematic drug discovery was provided. A number of practical examples were first described to illustrate the concepts and capabilities of image-based screening for drug and target discovery. Then, the prevalent bioimage informatics techniques, e.g., object detection, segmentation, tracking and visualization, were discussed. Subsequently, the widely used numerical features, phenotypes identification, classification, and profiling analysis were introduced to characterize the effects of drugs and targets. Finally, the major publicly available bioimage informatics software packages were listed for future reference. We hope that this review provided sufficient information and insights for readers to apply the approaches and techniques of bioimage informatics to advance their research projects.

## 8. Exercises

**Q1.** Understand the principle of using green fluorescent protein (GFP) to label the chromosome of HeLa cells.

**Q2.** Download a cellular image processing software package, then download some cell images, and use them as examples to perform the cell detection, segmentation, and feature extraction, and provide the analysis results.

**Q3.** Download a time-lapse image analysis software package, then download some time-lapse images, and use them as examples to perform cell tracking, and cell cycle phase classification, and provide the analysis results.

**Q4.** Download a neuron image analysis software package, then download some neuron images, and use them as examples to perform dendrite and spine detection, and provide the analysis results.

**Q5.** Implement the watershed and level set segmentation methods by using ITK functions (http://www.itk.org/) and test them on some cell images.

Answers to the Exercises can be found in Text S1.

## Supporting Information

Text S1 Answers to Exercises.
(DOCX)
