# Supplementary material for: Chapter 17: Bioimage Informatics for Systems Pharmacology
Source: PLoS Comput Biol. 2013 Apr 25;9(4):e1003043. doi: 10.1371/journal.pcbi.1003043 (PMC3635992; doi:10.1371/journal.pcbi.1003043)
Supplement: Text S1 — Answers to Exercises. (DOCX) [file pcbi.1003043.s001.docx]

**Exercises and Solutions**

**Q1.** Understand the principle of using green fluorescent protein (GFP) to label the chromosome of HeLa cells.

**Solution:** To investigate the dynamic chromosome appearance of Hela cells over time by using automated fluorescent microscopy, a method is needed to bind the GFP uniformly to chromosomes of HeLa cells so that GFP imaging indicates the spatial distribution (morphology appearance) of chromosomes within cells. To achieve this, GFP is required to bind to a protein that is able to uniformly localize to the chromosomes and will not affect the chromosome functions of HeLa cells. As histones are the principal structural proteins of eukaryotic chromosomes, they are widely used as the target protein bound with GFP to investigate the morphology of chromosomes within cells. For example, the human histone H2B protein was selected as the target protein binding with GFP. In brief, the cDNA encoding human H2B was tagged at its carboxyl terminus with DNA encoding codon-optimized enhanced GFP (H2B-GFP) and then transfected into HeLa cells. As a result, the HeLa cells could stably express the H2B-GFP protein that uniformly binds to chromosomes of HeLa cells such that dynamic morphology of chromosomes of HeLa cells could then be imaged by using automated fluorescent microscopy.

More details are provided here:

Kanda, T., K.F. Sullivan, and G.M. Wahl, Histone-GFP fusion protein enables sensitive analysis of chromosome dynamics in living mammalian cells. *Curr Biol*, 1998. 8(7): p. 377-85.

**Q2.** Download a cellular image processing software package, then download some cell images, and use them as examples to test the cell detection, segmentation, and feature extraction, and provide the analysis results.

**Solution:** Take CellProfiler software as an example.

s.2.1) Download and install the CellProfiler software from <http://www.cellprofiler.org/>

s.2.2) Download the multi-channel images and analysis pipeline of Drosophila Kc167 cells (Fruit fly cells) from <http://www.cellprofiler.org/examples.shtml>

s.2.3) Open CellProfiler, choose ‘File’ -> ‘Load pipeline’ to find the downloaded pipeline. Then a list of analysis modules, e.g. loadImages, IdentifyPrimaryObjects, IdentifySecondaryObjects, MeasureObjectIntensity, will be shown on the left of the CellProfiler interface.

s.2.4) Change the ‘Default Input Folder’ at the bottom of the CellProfiler interfacee to the file folder containing the downloaded example images.

s.2.5) Change the ‘Default Output Folder’ at the bottom of the CellProfiler interface to the file folder where you want to put the output results.

s.2.6) Click ‘Analyze images’ at the right bottom of the CellProfiler interface to run the analysis.

s.2.7) The CellProfiler software will show the segmentation results, and measurements of cells.

Try to add, remove or modify the analysis pipeline to check the analysis results. For more details, please refer to the on-line available documents of CellProfiler: <http://www.cellprofiler.org/>

**Q3.** Download a time-lapse image analysis software package, then download some time-lapse images, and use them as examples to test the cell tracking, and cell cycle phase classification, and provide the analysis results.

**Solution:** Take DCellIQ as an example.

For cell segmentation, tracking and cell cycle phase identification analysis:

s.3.1) Download the DCellIQ software package and sample images from: <http://www.cbi-tmhs.org/Dcelliq/downloading.html>

s.3.2) Download the example time-lapse image data of HeLa cells from: <http://www.cbi-tmhs.org/Dcelliq/downloading.html>

s.3.3) Run the DCellIQ in Matlab. Choose ‘File->GetDirectory’ to access the file folder containing the sample image data. Then choose ‘Image Processing -> Processing’. Input ‘yes’ in the popup window to do the cell tracking, feature extraction and classification with the default parameters.

s.3.4) Check the tracking results of individual cells by inputting ‘TraceID’ in the bottom of the DCellIQ interface, and check the cell cycle phase classification results with DCellIQUI.

For refining the cell cycle phase identification classifiers:

s.3.5) Run the DCellIQUI software to manually select and save 100 cells in interphase and metaphase.

s.3.6) Customize a SVM classifier in the DCellIQUI. First select the features you want to use by clicking the ‘selection feature’ radio button and then click the ‘Offline SVM -> Offline SVM training’ button to train your own SVM classifier.

s.3.7) Click the ‘Offline SVM -> Phase Identification (offline)’ to categorize the rest of cells into their corresponding cell cycle phases.

For the more details, please refer to the user manual of DCellIQ.

For the SVM classifier, the LibSVM libraries are used. Please find more information about the usage of SVM classifier at: <http://www.csie.ntu.edu.tw/~cjlin/libsvm/>

**Q4.** Download a neuron image analysis software package, then download some neuron images, and use them as examples to perform the dendrite and spine detection, and provide the analysis results.

**Solution:** Take NeuronStudio as an example.

s.4.1) Download the NeuronStudio from: <http://research.mssm.edu/cnic/tools-ns.html>

s.4.2) Download the example neuron image data, e.g. “Sample dataset, model, and spines #2” from: <http://research.mssm.edu/cnic/tools.html>

s.4.3) Run the NeuronStudio software, and load in the examples images by choosing ‘File’ -> ‘Open’

s.4.4) Detect the neuron dendrite by following the guide:

<http://research.mssm.edu/cnic/help/ns/trace.html>

s.4.5) Detect the neuron spine by following the guide:

<http://research.mssm.edu/cnic/help/ns/spines.html>

For more details, please refer to the on-line documents of NeuronStudio:

<http://research.mssm.edu/cnic/help/ns/index.html>

**Q5.** Implement the watershed and level set segmentation methods by using ITK (<http://www.itk.org/>) and test them on some cell images.

**Solution:** Before implementing the image analysis algorithms using ITK, make sure you have correctly installed the ITK, cmake and a C++ compiler (e.g. Microsoft Visual Studio). If not, please install them using the following links: ITK: <http://www.itk.org/> and cmake: <http://www.cmake.org/>

1. Implement the watershed segmentation example in ITK.

s.5.a.1) Download the two files: “WatershedImageFilter.cxx” and “CMakeLists.txt” from: <http://www.itk.org/Wiki/ITK/Examples/Segmentation/WatershedImageFilter>

s.5.a.2) Generate a c++ project by applying the cmake to the two files.

s.5.a.3) Generate the executable watershed segmentation program by compiling the generated c++ project.

b) Implement the level set segmentation example in ITK.

s.5.b.1) Download the two files:

“SinglephaseChanAndVeseDenseFieldLevelSetSegmentation.cxx” and “CMakeLists.txt” from:

<http://www.itk.org/Wiki/ITK/Examples/Segmentation/SinglephaseChanAndVeseDenseFieldLevelSetSegmentation>

s.5.b.2) Generate a c++ project by applying the cmake to the two files.

s.5.b.3) Generate the executable watershed segmentation program by compiling the generated c++ project.

The previously downloaded sample images could be used to test these two segmentation methods.
